# Pregnancy Outcome following Prenatal Diagnosis of Chromosomal Anomaly: A Record Linkage Study of 26,261 Pregnancies

**DOI:** 10.1371/journal.pone.0166909

**Published:** 2016-12-01

**Authors:** Myrthe Jacobs, Sally-Ann Cooper, Ruth McGowan, Scott M. Nelson, Jill P. Pell

**Affiliations:** 1 Institute of Health and Wellbeing, University of Glasgow, Glasgow, United Kingdom; 2 West of Scotland Regional Genetics Service, Glasgow, United Kingdom; 3 School of Medicine, University of Glasgow, Glasgow, United Kingdom; University of Warwick, UNITED KINGDOM

## Abstract

Previous studies have demonstrated the influence of changes in the age at which women give birth, and of developments in prenatal screening and diagnosis on the number of pregnancies diagnosed and terminated with chromosomal anomalies. However, we are unaware of any population studies examining pregnancy terminations after diagnosis of chromosomal anomalies that has included all aneuploidies and the influence of maternal factors. The aims of this study were to examine the association between results of prenatal tests and pregnancy termination, and the proportion of foetuses with and without chromosomal anomalies referred for invasive diagnostic tests over time. Diagnostic information of 26,261 prenatal invasive tests from all genetic service laboratories in Scotland from 2000 to 2011 was linked to Scottish Morbidity Records to obtain details on pregnancy outcome. Binary logistic regression was carried out to test the associations of year and type of diagnosis with pregnancy termination, while controlling for maternal age, neighbourhood deprivation and parity. There were 24,155 (92.0%) with no chromosomal anomalies, 1,483 (5.6%) aneuploidy diagnoses, and 623 (2.4%) diagnoses of anomaly that was not aneuploidy (including translocations and single chromosome deletions). In comparison with negative test results, pregnancies diagnosed with trisomy were most likely to be terminated (adjusted OR 437.40, 95% CI 348.19–549.46) followed by other aneuploid anomalies (adjusted OR 95.94, 95% CI 69.21–133.01). During the study period, fewer pregnancies that were diagnosed with aneuploidy were terminated, including trisomy diagnoses (adjusted OR 0.44, 95% CI 0.26–0.73). Older women were less likely to terminate (OR 0.35, 95% CI 0.28, 0.42), and parity was also an independent predictor of termination. In keeping with previous findings, while the number of invasive diagnostic tests declined, the proportion of abnormal results increased from 6.09% to 10.88%. Systematic advances in prenatal screening have improved detection rates for aneuploidy. This has been accompanied by a reduction in the rate of termination for aneuploidy. This may reflect societal changes with acceptance of greater diversity, but this is speculation, and further research would be needed to test this.

## Introduction

The age at which women give birth has increased progressively in many high income countries, such that in Scotland, the proportion of mothers giving birth at age 35 or over has increased from 14.9% in 2000 to 19.7% in 2011 [1]. Reflecting the recent changes in maternal demographics and that maternal age increases the likelihood of foetal aneuploid chromosomal anomalies, including Edwards, Patau, Klinefelter and Triple X syndromes, and the commonest type, Down syndrome [2], the number of aneuploid pregnancies has increased [3, 4]. Detection rates have also increased, as prenatal screening and diagnostic techniques for chromosomal anomalies have become more widely available and more sensitive [5–7]. In England and Wales, among mothers younger than 37 years the proportion of prenatal aneuploid diagnoses increased from 3% in 1989 to 43% in 2008, whilst this proportion remained at 70% for mothers aged 37 or older [3]. Similar trends have been reported in Victoria, Australia and in Denmark [8, 9].

Recent systematic reviews of factors, which may influence the decision to terminate or continue the pregnancy, have reported inconsistent results. With respect to maternal age, studies have reported increased likelihood of termination for Down syndrome in both younger and older women, as well as no association with age [10, 11]. Similarly, a systematic review on decisions to terminate pregnancies following diagnosis of sex chromosome abnormalities found no relationship with maternal age [12]. Two studies examined this for a wider range of chromosome abnormalities [13, 14]. While Drugan et al. (1990) found no difference in the age of women who did and did not terminate [13], Shaffer et al., (2006) concluded that the effect of age may be dependent on the diagnosis; with older women being more likely to terminate a pregnancy affected by trisomy but less likely to terminate after a diagnosis of sex chromosome abnormality [14].

With respect to parity, an association has been demonstrated between parity and the decision to terminate for Down syndrome [15] but the results are inconsistent in relation to termination decisions for sex chromosome anomalies [12]. Two studies examined previous obstetric history for a wider range of chromosome abnormalities and found that while the number of previous livebirths and spontaneous abortions did not differ between women who did and did not terminate, history of previous terminations increased the likelihood of termination of the current pregnancy [13, 16].

Whilst the evidence in relation to maternal characteristics and past obstetric history is inconclusive, the actual diagnosis has been found to be more clearly related to the decision to terminate. Trisomy, including Down syndrome, is associated with higher termination rates than sex chromosome abnormalities [14, 16–18]. Furthermore, of the sex chromosome abnormalities, Turner syndrome and Klinefelter syndrome have higher termination rates than Triple X or 47,XYY [12]. Abnormal ultrasound findings identified either before or after the diagnosis of chromosome abnormalities (including trisomy, sex chromosome abnormalities, and balanced and unbalanced translocations) have been found to further increase the likelihood of pregnancy termination [13, 19].

In spite of increasing maternal age and increasing detection rates of chromosomal anomalies, termination rates have been reported as remaining stable over the last decade in both single centre and population studies [3, 8, 14]. However, we are unaware of any population study that has included all aneuploidies and studied the influence of maternal factors and specific prenatal diagnoses on termination rates. To address this, we used Scotland wide data to examine whether there have been changes over time in the proportion of pregnancies affected with and without chromosomal anomalies who are referred for invasive diagnostic tests and the proportion of pregnancies associated with chromosomal anomalies that are terminated. We also examined whether termination rates differed by prenatal diagnosis.

## Materials and Methods

### Data sources

The study cohort comprised all pregnancies during which genetic testing had been undertaken from January 2000 to December 2011 inclusive, in four NHS regional genetic services (Glasgow, Edinburgh, Aberdeen, Dundee), covering the total population in Scotland. A service was also provided in Inverness until 2010 when Aberdeen took over its cases. Chorionic villus sampling or amniocentesis (depending upon gestational stage) was used to collect foetal cells for prenatal diagnosis via karyotyping, fluorescence in situ hybridization (FISH), or quantitative fluorescence polymerase chain reaction (QF-PCR). Reasons for referral to genetic counselling services included advanced maternal age, an abnormal ultrasound scan, a high-risk biochemistry result, family history of chromosomal abnormalities and maternal anxiety about having an abnormal pregnancy. Ultrasound scans and biochemistry tests are part of the pregnancy screening that is offered to all pregnant women in Scotland.

The screening programmes offered in Scotland were standardised from 2001 to a second trimester blood sample test for Down syndrome and neural tube defects using two serum markers [20]. Further changes were made in 2008 [21] to a first trimester screen for Down syndrome comprising blood biomarkers combined with ultrasound measurement of foetal nuchal translucency; a second trimester foetal anomaly ultrasound examination; and a second trimester screen with quadruple markers, for those women who did not present early enough for the first trimester screening. In 2011, the cut off for a high-risk screening result was changed to 1 in 150 from 1 in 250 for the combined first trimester screen and from 1 in 220 for the second trimester quadruple screening. This was in order to achieve a sensitivity of greater than 90% and a screen positive rate (i.e. the total percentage of abnormal results) of less than 2% for the combined first trimester screen, and a sensitivity of more than 75% and a screen positive rate of less than 3% for the second trimester quadruple screening [22]. Women with screening results indicative of high risk are referred for further invasive diagnostic tests, i.e. chorionic villus sampling or amniocentesis.

Data were provided by all the genetic service laboratories in Scotland. These included the month and year of investigation, diagnostic test results, and pregnancy outcome if known. Year of investigation was categorised into: 2000–2003, 2004–2007 and 2008–2011. Diagnoses were grouped into three main categories: aneuploidy, other anomaly, and normal. Aneuploidy comprised Down syndrome, Edwards syndrome, Patau syndrome, Turner syndrome, Klinefelter syndrome, triple X, 47 XYY, triploidy and tetraploidy. In the secondary analyses, the aneuploid karyotypes were further categorised into two sub-groups: trisomy 21, 18, or 13 (Down syndrome, Edwards syndrome, Patau syndrome, but not triple X nor XXY) and other aneuploid anomalies (Turner syndrome, Klinefelter syndrome, Triple X, 47 XYY, Triploidy and Tetraploidy). The ‘other anomaly’ category consisted of all other abnormal results, including balanced and unbalanced (Robertsonian and reciprocal) translocations, and single chromosome deletions, inversions and variants.

The data from the genetic service laboratories were linked, at an individual level, using the Community Health Index (a unique personal identifier) to two Scotland-wide health administrative databases routinely collected and centrally collated as part of the Scottish Morbidity Record (SMR). SMR01 collects data on all acute hospital admissions and day cases including disease codes and the SMR02 maternity records collect data on all discharges from obstetric units in Scotland, including details of live births and stillbirths, and maternal age, marital status, parity, and the Scottish Index of Multiple Deprivation (SIMD) [23].

Permission to access, link and analyse these data was granted by the Privacy Advisory Committee (PAC) to the National Health Service (NHS) National Services Scotland (NSS) and the Registrar General. Written informed consent was not obtained from participants for their clinical records to be used in this study, but patient records were anonymized and de-identified prior to analysis.

### Data

SIMD is an area-based measure of socioeconomic deprivation derived for postcode of residence (mean population 800) using Census data on 7 domains: employment, income, health, education, access to services, crime, and housing [23]. The index is categorised into quintiles for the general population ranging from 1 (least affluent) to 5 (most affluent). The maternal postcode of residence, recorded on SMR01 or SMR02, is then used to allocate the mother and her offspring to an SIMD quintile. Maternal age was provided in years and coded into 5 categories: <25, 25–29, 30–34, 35–39, ≥40. Marital status was categorised as married, never married, widowed/ divorced, and not known. Parity was defined as the number of previous pregnancies resulting in a live birth or stillbirth and coded into three categories: 0, 1, and ≥ 2.

SMR02 records provide pregnancy outcome as livebirth and stillbirth (i.e. baby born dead after 24 weeks gestation), and distinguish between therapeutic abortion, spontaneous, incomplete and missed abortion (i.e. baby dies before 24 weeks gestation), trophoblastic disease, and other and unspecified abortion. SMR01 records contain ICD-10 disease codes and were used to identify any therapeutic abortions (O04) and spontaneous, incomplete and missed abortions (O02-03) in women without an SMR02 record. Other abortions, unspecified abortions, trophoblastic disease and other abnormal products of conception were grouped as ‘other outcomes’. The remaining outcomes were then categorised into pregnancies ending in therapeutic abortion, and those that did not. The latter included livebirths, stillbirths, and spontaneous, incomplete and missed abortions.

### Statistical analyses

Multiple pregnancies were identified and excluded. Cross tabulations and Pearson’s chi-squared tests were employed to compare the characteristics of the prenatal diagnostic groups. Binary logistic regression was undertaken to determine whether year and diagnosis were associated with termination of pregnancy and whether any associations were independent of maternal age, parity and SIMD. Interactions of year with maternal age, parity and SIMD were also examined. Results are presented as unadjusted and adjusted odds ratios with 95% confidence intervals (CI). All analyses were carried out in SPSS 22.

## Results

Data were available on 26,594 prenatal diagnostic tests. Following exclusion of 281 multiple pregnancies and 52 where the diagnostic result was not recorded, the study sample comprised 26,261 pregnancies. The distribution across the genetic laboratories reflected the sizes of their catchment populations: Glasgow 43.4%; Edinburgh 26.9%; Aberdeen 14.0%; Tayside 12.0%; and Highland 3.6%. The most common reason for referral to the genetic services was a high risk screening result: high risk biochemistry 57.8%; and an abnormal scan (11.6%). Other reasons included: advanced maternal age 20.1%; family history of chromosomal abnormalities 6.6%; maternal anxiety 2.8%; and in-vitro fertilization 1.1%. Overall, 24,155 (92.0%) tests revealed no detectable chromosomal anomaly, 1,483 (5.6%) aneuploidy, and 623 (2.4%) another anomaly (a very heterogenous group, of which 111 were balanced translocations, 51 Robertsonian translocations, and a number of single chromosomal deletions and inversions). Aneuploidy included 851 (57.4%) with Down syndrome, 302 (20.4%) Edwards syndrome, 93 (6.3%) Patau syndrome, 122 (8.2%) Turner syndrome, 25 (1.7%) Klinefelter syndrome, 17 (1.1%) triple X, 8 (0.5%) 47XYY, and 65 (4.4%) triploidy or tetraploidy. The characteristics of patients differed significantly according to the prenatal diagnosis (Table 1). The proportion of tests with a positive result increased over time (Table 1). Overall, anomalies were more common among mothers who were younger, less affluent, and who had lower parity. In contrast, among the sub-group diagnosed with aneuploidy, trisomy 21, 18 or 13 was more common among mothers who were older, more affluent, married, and who had higher parity.

**Table 1:** Characteristics of study participants by test result.

	All tests						Aneuploidy					
	Normal (n = 24,155)		Aneuploidy (n = 1483)		Other anomaly (n = 623)		P-value*	Trisomy^1^ (n = 1,246)		Other aneuploid anomaly^2^ (n = 237)		P-value*
	n	%	n	%	n	%		n	%	n	%	
**Year of test**												
2000–2003	8,810	93.7	402	4.3	188	2.0	<.001	331	82.3	71	17.7	.256
2004–2007	8,264	91.8	539	6.0	197	2.2		452	83.9	87	16.1	
2008–2011	6,957	90.1	534	6.9	232	3.0		455	85.2	79	14.8	
Missing	124		8		6			8		0		
**Maternal age (years)**												
<25	1,285	90.0	93	6.5	49	3.4	<.001	57	61.3	36	38.7	<.001
25–29	1,981	90.4	130	5.9	81	3.7		82	63.1	48	36.9	
30–34	4,941	93.0	233	4.4	138	2.6		188	80.7	45	19.3	
35–39	9,445	94.4	398	4.0	166	1.7		357	89.7	41	10.3	
≥40	4,720	92.5	294	5.8	89	1.7		267	90.8	27	9.2	
Missing	1,783		335		100			295		40		
**SIMD quintile**												
1 (least affluent)	2,926	92.2	168	5.3	80	2.5	.045	121	72.0	47	28.0	<.001
2	3,429	92.8	187	5.1	80	2.2		148	79.1	39	20.9	
3	4,228	93.8	203	4.5	78	1.7		174	85.7	29	14.3	
4	5,427	93.4	273	4.7	111	1.9		233	85.3	40	14.7	
5 (most affluent)	6,287	92.9	312	4.6	171	2.5		270	86.5	42	13.5	
Missing	1,858		340		103			300		40		
**Marital status**												
Married	10,377	93.2	524	4.7	235	2.1	.550	449	85.7	75	14.3	.001
Never married	4,196	92.5	235	5.2	103	2.3		176	74.9	59	25.1	
Widowed/divorced	1,271	92.6	74	5.4	27	2.0		62	83.8	12	16.2	
Missing	8,311		650		258			559		91		
**Parity**												
0	7,751	92.4	417	5.0	219	2.6	.006	328	78.7	89	21.3	.003
1	8,218	93.5	403	4.6	168	1.9		337	83.6	66	16.4	
≥2	6,152	93.3	315	4.8	126	1.9		274	87.0	41	13.0	
Missing	2,034		348		110			307		41		

n, number; SIMD, Scottish Index of Multiple Deprivation.

^1^Down, Edwards and Patau syndrome.

^2^Turner syndrome, Klinefelter syndrome, Triple X, 47 XYY, Triploidy and Tetraploidy.

*p values were calculated using the χ^2^ test; χ^2^ for trend test was used for 2*n ordinal data.

Table 2 contains the results of the binary logistic regression models undertaken to examine changes in the use of termination over time. Overall, terminations became significantly more common over the three time periods: 2004–2007 (OR 1.26, 95% CI 1.11–1.43, p<0.001) and 2008–2011 (OR 1.35, 95% CI 1.19–1.53, p<0.001) in comparison to 2000–2003 (p < .001 for overall effect). However, there were significant interactions between year of diagnosis and type of diagnosis, maternal age, SIMD quintile and parity (all p<0.001). On sub-groups analysis (Table 2), terminations decreased over time following diagnosis of aneuploidy even after adjustment for potential confounders, but increased significantly for other anomalies. For the women whose pregnancy did not have a chromosomal anomaly, terminations rates were 1.7% in 2000–2003, 2.1% in 2004–2007, and 2.0% in 2008–2011.

**Table 2:** Binary Logistic Regression for the Association between Year and Termination for the Full Sample.

		Univariate				Multivariate^1^			
		2000–2003	2004–2007 OR (95% CI) P-value	2008–2011 OR (95% CI) P-value	P-value	2000–2003	2004–2007 OR (95% CI) P-value	2008–2011 OR (95% CI) P-value	P-value
Diagnosis	Normal chromosomes	1.00	1.26 (1.00–1.59) .047	1.24 (0.97–1.58) .081	.097	1.00	1.32 (1.02–1.72) .043	1.21 (0.91–1.60) .186	.122
	Aneuploidy	1.00	0.54 (0.37–0.79) .002	0.55 (0.37–0.81) .003	.003	1.00	0.50 (0.33–0.74) .001	0.57 (0.38–0.86) .007	.002
	Other anomaly	1.00	1.83 (1.10–3.04) .019	2.04 (1.25–3.33) .004	.012	1.00	2.33 (1.30–4.16) .004	2.62 (1.50–4.58) .001	.002
Age	<35	1.00	1.20 (0.99–1.46) .062	1.20 (0.98–1.45) .074	.109	1.00	1.15 (0.89–1.48) .293	1.12 (0.86–1.45) .400	.538
	≥35	1.00	1.39 (1.14–1.68) .001	1.66 (1.37–2.02) < .001	<.001	1.00	0.99 (0.72–1.36) .940	1.08 (0.78–1.49) .663	.856
SIMD	1 (least affluent)	1.00	1.07 (0.75–1.54) .694	1.28 (0.90–1.81) .167	.352	1.00	0.79 (0.49–1.27) .333	1.04 (0.66–1.63) .859	.454
	2	1.00	1.49 (1.06–2.09) .021	1.74 (1.24–2.43) .001	.005	1.00	1.25 (0.77–2.05) .365	1.38 (0.86–2.24) .187	.409
	3	1.00	0.90 (0.64–1.26) .539	1.05 (0.75–1.45) .791	.681	1.00	0.86 (0.52–1.43) .561	0.95 (0.58–1.57) .842	.841
	4	1.00	1.06 (0.80–1.42) .671	1.33 (1.00–1.77) .051	.123	1.00	1.10 (0.71–1.71) .679	0.89 (0.56–1.40) .604	.659
	5 (most affluent)	1.00	1.76 (1.37–2.27) < .001	1.65 (1.26–2.18) < .001	<.001	1.00	1.44 (0.98–2.12) .066	1.41 (0.93–2.12) .105	.136
Parity	0	1.00	1.36 (1.10–1.69) .005	1.32 (1.05–1.64) .015	.012	1.00	1.27 (0.93–1.73) .136	1.19 (0.87–1.64) .281	.310
	1	1.00	1.44 (1.15–1.82) .002	1.46 (1.15–1.86)	.002	1.00	1.33 (0.94–1.87) .110	1.29 (0.91–1.84) .159	.222
	≥2	1.00	0.89 (0.67–1.18) .409	1.57 (1.21–2.05) .001	<.001	1.00	0.66 (0.43–1.00) .049	0.88 (0.59–1.32) .543	.135

OR odds ratio; CI confidence interval; SIMD, Scottish Index of Multiple Deprivation.

^1^Adjusted for Diagnosis, Maternal age, SIMD quintiles, Parity.

When the univariate model was re-run including only pregnancies in which trisomy was diagnosed, termination rates fell significantly over time: 2004–2007 (OR 0.44, 95% CI 0.27–0.72, p = 0.001) and 2008–2011 (OR 0.41, 95% CI 0.25–0.67, p<0.001) in comparison to 2000–2003 (p = .001 for overall effect). Adjustment for maternal age, SIMD and parity did not alter the results: 2004–2007 (adjusted OR 0.40, 95% CI 0.24–0.66, p<0.001) and 2008–2001 (OR 0.44, 95% CI 0.26–0.73, p = 0.002) in comparison to 2000–2003 (p = .001 for overall effect). There were no statistically significant interactions.

The percentage of pregnancies that were terminated varied significantly by diagnosis: 85.2% for trisomy, 65.4% for other aneuploid anomalies and 1.9% for normal karyotypes (χ^2^ (2) = 13283.00, p < .001). After adjustment for year of testing, maternal age, SIMD quintile and parity, diagnosis remained a significant predictor of termination. In comparison with normal chromosomal test results, termination was significantly most likely following a diagnosis of trisomy 21, 18, or 13 (adjusted OR 437.40, 95% CI 348.19–549.46, p<0.001) and other aneuploidy anomalies (adjusted OR 95.94, 95% CI 69.21–133.01, p<0.001). Older women were less likely to terminate their pregnancy (OR 0.35, 95% CI 0.28, 0.42). Parity was a significant independent predictor of termination (Table 3)

**Table 3:** Independent predictors of pregnancy outcomes.

Predictor	Univariate			Adjusted^1^		
	Odds ratio	95% CI	P-value	Odds ratio	95% CI	p-value
**Diagnosis**			<.001			<.001
Normal chromosomes	1			1		
Aneuploidy	304.50	250.98–369.44	<.001	437.40	348.19–549.46	<.001
Other anomaly	97.00	72.43–129.91	<.001	95.94	69.21–133.01	<.001
**Year**			<.001			.972
2000–2003	1			1		
2004–2007	1.27	1.12–1.44	<.001	0.98	0.79–1.22	.874
2008–2011	1.37	1.20–1.55	<.001	0.97	0.78–1.22	.815
**Age**						
<35	1			1		
≥35	0.59	0.53–0.65	<.001	0.35	0.28–0.42	<.001
**SIMD**			.001			.221
1 (least affluent)	1			1		
2	0.98	0.81–1.18	.822	1.24	0.91–1.70	.174
3	0.73	0.60–0.88	.001	0.88	0.64–1.21	.434
4	0.77	0.64–0.92	.003	0.99	0.73–1.34	.934
5 (most affluent)	0.84	0.71–0.99	.040	1.10	0.82–1.47	.538
**Parity**						.037
0	1			1		
1	0.81	0.72–0.92	.001	0.86	0.70–1.06	.159
≥2	0.79	0.69–0.90	.001	0.73	0.58–0.93	.011

CI confidence interval; SIMD Scottish Index of Multiple Deprivation.

^1^Adjusted for diagnosis, year of testing, maternal age, SIMD quintiles, parity

### Ascertainment of prenatal screening

**Fig 1:**
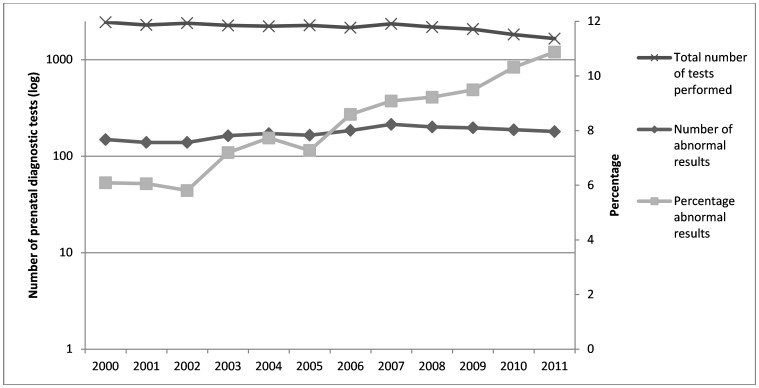
Ascertainment of prenatal screening.

Between 2000 and 2011, the total number of invasive diagnostic tests carried out each year decreased from 2,447 to 1,655. However, the number of diagnoses made each year of aneuploidy and other anomalies has increased gradually from 149 to 180 (see Fig 1), resulting in an increase in the percentage of all abnormal karyotypes detected amongst those who were referred for diagnostic testing from 6.09% to 10.88%. This indicates that, over the study period, the positive predictive value of screening procedures has improved as fewer foetuses with normal karyotypes were considered as high risk and referred for prenatal diagnostic testing.

## Discussion

### Principal findings

Overall, the likelihood of a diagnosis of aneuploidy resulting in a decision to terminate the pregnancy has fallen over time. However, the use of termination varies greatly within the aneuploidy group. A diagnosis of trisomy is more likely to result in termination, perhaps due to the more serious health implications. It is likely that the temporal trend we observed predates our study period, as our trisomy pregnancy termination rate of 85% compares with a previous Scottish report in the 1990s of 97.3% for Down syndrome, 85.9% for trisomy 18, and 90.0% for trisomy 13 [24]. Our results may indicate that society is becoming more accepting of diversity and people with disabilities, or that support is more readily available for affected parents and offspring. People with Down syndrome are considerably more visible in Scottish society now than in the era of the long-stay intellectual disabilities hospitals. However, this interpretation is of course purely speculative, and a study using qualitative research methods would be necessary to test if these factors explain the trend we have identified. Conversely, although rarer disorders, terminations for other (non-aneuploidy) anomalies have increased over the study period. These other conditions, such as balanced and unbalanced translocations, single chromosome deletions, inversions and variants may have milder, more variable and less certain phenotypes compared to the aneuploidies, and it is possible that this influences decision-making on termination.

Previous research provided the contrary findings that termination rates were stable over time for aneuploid diagnoses in Australia, England and Wales, and in the USA [3, 8, 14]. However, these studies covered earlier time periods, namely the 1980s, 1990s, and early 2000s, and patterns of termination may differ between countries. Indeed, the termination rate we report for the period of 2000–2011 of 85.2% for trisomy pregnancies is lower than the latest rates in England and Wales of 90.1% for Down syndrome, 92.7% for Edwards syndrome and 90.3% for Patau syndrome [25].

We found that older women were markedly less likely to terminate, and also found that parity was an independent predictor of termination, whereas neighbourhood deprivation was not.

Religion is likely to play a role in decisions around pregnancy termination [10, 26]. However, the Census in Scotland shows that the proportion of the population that identified themselves as not religious increased from 28% in 2001 to 37% in 2011 [27, 28]. The counselling process after a diagnosis may also impact on the decision to terminate [12, 16]. In a systematic review focussing on termination decisions after a diagnosis of a sex chromosome abnormality, Jeon et al. (2012) found that women who were counselled by non-geneticists were more likely to terminate the pregnancy and those who were counselled by a genetic specialist were less likely to terminate [12]. In Scotland counselling is provided by obstetricians, frequently with the input of clinical genetics. In addition, those who experienced more directive counselling were more likely to terminate, and those who experienced less directive counselling were less likely to terminate. Uptake of a second genetic counselling session was related to a lower likelihood to terminate the pregnancy following diagnosis of trisomy or sex chromosome abnormality [16].

A number of studies have found a relationship between earlier gestation at diagnosis and greater likelihood to terminate the pregnancy [11, 15]. Drugan et al. (1990) explained that termination later in the pregnancy may be more difficult as bonding may be stronger and pressures from society, family and friends may influence the decision [13]. Earlier in the pregnancy, the decision to terminate may be more private and the process may be less emotionally damaging. The introduction of first trimester screening in Scotland in 2008 may have decreased the average gestational age at which women receive a prenatal diagnosis, however, we did not test this in this study.

In keeping with previous reports, pregnancies diagnosed with trisomy remained more likely to be terminated than pregnancies diagnosed with other aneuploid anomalies [14, 16–18]. Also in keeping with previous findings, our study also shows that the total number of invasive diagnostic tests decreased over the study period, while the proportion of those undertaken that had an abnormal result increased. This shows that the changes to prenatal screening made in Scotland have improved the positive predictive value of prenatal screening.

### Strengths and weaknesses

This is the first non-selective, population based study in Scotland examining pregnancy terminations after prenatal diagnosis of chromosomal anomalies. It took account of potential confounders such age, SIMD and parity. It was not subject to selection bias, as the study was based on routine data sources from all genetic service laboratories in Scotland. We had access to both SMR-02 maternity records and SMR-01 hospital admissions to ascertain pregnancy outcome, thereby improving completeness. The outcome could not be determined for only 7.3% of the pregnancies. This is likely to be because of admission to a hospital outside of Scotland or the NHS.

In order to protect anonymity, the current study could not report data on individual diagnostic groups, as the groups were too small. There was a large amount of missing data for marital status which was therefore not controlled for in the analyses. Marital status however is closely related to age and SIMD, which were both included. In addition, we did not have access to information on religion. Years were collapsed into groups of three to avoid having too many predictors and reduced power, especially for analyses that excluded normal karyotypes. Therefore, detailed information regarding year to year changes over time may have been lost.

### Conclusions

In Scotland, termination rates for trisomy are higher than for other aneuploidy, but rates for both have fallen over time. This may be linked to societal changes in accepting greater diversity, but further research would be needed to test this. Older women were less likely to terminate. In keeping with previous findings, screening procedures are now better at identifying those pregnancies in which the diagnosis will be positive therefore fewer pregnancies with normal karyotypes are undergoing prenatal invasive diagnostic tests, which have a risk of procedure related miscarriage. Recent developments in non-invasive prenatal testing may improve this further.
